# The stability of aluminium oxide monolayer and its interface with two-dimensional materials

**DOI:** 10.1038/srep29221

**Published:** 2016-07-06

**Authors:** Ting Ting Song, Ming Yang, Jian Wei Chai, Martin Callsen, Jun Zhou, Tong Yang, Zheng Zhang, Ji Sheng Pan, Dong Zhi Chi, Yuan Ping Feng, Shi Jie Wang

**Affiliations:** 1 Institute for Structure and Function and Department of Physics, Chongqing University, Chongqing, 400044 People’s Republic of China; 2 Institute of Materials Research and Engineering, A^*^STAR (Agency for Science, Technology and Research), #08-03, Innovis, 2 Fusionopolis Way, 138634 Singapore; 3 Centre for Advanced 2D Materials and Graphene Research, National University of Singapore, 6 Science Drive 2, 117546 Singapore; 4 Department of Physics, National University of Singapore, 2 Science Drive 3, 117542 Singapore

## Abstract

The miniaturization of future electronic devices requires the knowledge of interfacial properties between two-dimensional channel materials and high-*κ* dielectrics in the limit of one atomic layer thickness. In this report, by combining particle-swarm optimization method with first-principles calculations, we present a detailed study of structural, electronic, mechanical, and dielectric properties of Al_2_O_3_ monolayer. We predict that planar Al_2_O_3_ monolayer is globally stable with a direct band gap of 5.99 eV and thermal stability up to 1100 K. The stability of this high-*κ* oxide monolayer can be enhanced by substrates such as graphene, for which the interfacial interaction is found to be weak. The band offsets between the Al_2_O_3_ monolayer and graphene are large enough for electronic applications. Our results not only predict a stable high-*κ* oxide monolayer, but also improve the understanding of interfacial properties between a high-*κ* dielectric monolayer and two-dimensional material.

Two-dimensional (2D) materials present exceptional electronic, optical, mechanical, and thermal properties, as well as a high surface/volume ratio, all of which make them promising for applications ranging from electronics to energy storage. The discovery of graphene, a single layer of carbon atoms arranged in planar honeycomb lattices, has stimulated dramatic research interest on other 2D materials in order to realize novel functionalization and also further miniaturization of electronic devices^1^,^2^,^3^,^4^,^5^,^6^. Analogously to graphene, many other 2D materials have been exfoliated from their corresponding constituents’ layered crystal structures, which feature strong intra-layer covalent bonding and weak inter-layer binding dominated by van der Waals interaction^7^,^8^,^9^, *i.e.* BN^10^, transition metal dichalcogenide monolayers^11^,^12^, group IV and group III metal chalcogenide^13^,^14^,^15^, and phospherene^16^,^17^. Buckled 2D materials such as silicene, germanene, and stanene^18^,^19^,^20^,^21^ have been reported to be stable, while the interaction between layers in their parent bulk materials are dominated by the formation of *sp*^3^ covalent bonds. Furthermore, 2D structures based on binary compounds of the group IV or group III–V elements, have been predicted to be stable by first-principles calculations^22^,^23^,^24^,^25^, whose structures can be either planar, buckled, or low buckled, depending on the corresponding atomic radius and electronegativity. In addition, first-principles calculations also suggest that the 2D compounds consisting of heavy atoms such as PbI_2_^26^ and BiI_3_^27^ are stable.

More recently, many efforts have been made to realize oxide based crystalline 2D sheets. For example, a stoichiometric silica 2D sheet with bi-layer tetrahedral configuration has been grown using a Ru (0001) substrate as the support^28^,^29^, and various possible 2D structures of SiO_2_ have been proposed by first-principles calculations^30^. It has been reported that by using sputtering a 2D phase of TiO_2_ with desirable reduced band gap can be formed on a rutile TiO_2_ (011) surface, for which the corresponding atomic structure has been refined by subsequent theoretical studies^31^,^32^,^33^. Stable 2D high-*κ* dielectric monolayers have also been found. A Y_2_O_3_ monolayer with a hexagonal lattice has been deposited on graphene^34^. MgO (111) single layer has been grown on yttrium stabilized zirconia (111) substrate^35^. Moreover, transition metal oxide nanosheets, including ZnO, TiO_2_, Co_3_O_4_, and WO_3_, have been synthesized by rationally employing lamellar reverse micelles method^36^. All these findings motivate us to explore other high-*κ* dielectrics based 2D structures. Al_2_O_3_ is an excellent electrical insulator, which has been widely used in current electronic devices. Thus, it is highly desired to explore the stability of its 2D form, and understand the related physical properties. In this study, *via* density-functional theory (DFT) based first-principles calculations, we predict that planar Al_2_O_3_ monolayer with a hexagonal lattice is stable. This oxide monolayer is found to be an insulator with a direct gap of 5.99 eV and a dielectric constant of 2.51. It is further found that the interaction between Al_2_O_3_ monolayer and graphene is weak, which leads to band offsets larger than 1 eV.

## Method

The particle swarm optimization (PSO) method implemented in the CALYPSO^37^,^38^ program has been used to search the potential stable structures of Al_2_O_3_ monolayer with the constrained stoichiometric formula of Al_2_O_3_. This method has been used to predict a number of 2D materials successfully^39^. In this PSO simulation, the number of formula units per simulation cell, the number of generations (the number of CALYPSO steps), and the population size (the number of configurations in each step) were set to be 4, 40, and 30, respectively. Among the structures obtained by the PSO method, the 2D planar structure with hexagonal lattice is the most energetically favorable.

First-principle calculations, using DFT based VASP code^40^,^41^, were carried out to optimize the structure and to study electronic, mechanical, and optical properties of the predicted planar structure. The generalized gradient approximation (GGA) in the form of Perdew-Burke-Ernzerhof (PBE) was used for the exchange-correlation functional, and the projector augmented wave (PAW) potentials were selected to describe the interaction between electrons and ions^42^,^43^. The electronic wave functions were expanded with a cutoff energy of 500 eV. The first Brillouin zone of Al_2_O_3_ monolayer, *α*-Al_2_O_3_ bulk, and hybrid structures of Al_2_O_3_ monolayer on the substrates of graphene and Al (111) was sampled by Γ centered 18 × 18 × 1, 12 × 12 × 4, 6 × 6 × 1, and 9 × 9 × 1 *κ*-point meshes, respectively. A vacuum layer with 15 Å thickness has been inserted normal to the Al_2_O_3_ surface for all slab structures to minimize the interaction between neighboring image surfaces. In order to get an improved electronic structure, the Heyd-Scuseria-Ernzerhof hybrid functionals (HSE06) were used^44^. The van de Waals (vdW) effects were included for the hybrid structures using Grimme’s DFT-D3 method^45^. The criteria for the convergence of electronic and ionic optimization for all calculations were set to 10^−6^ eV and 0.01 eV/Å, respectively. To explore thermal stability of Al_2_O_3_ monolayer, *ab initio* molecular dynamics (MD) simulations were performed with a time step of 1 fs, canonical ensemble (NVT), and Nos*é* heat bath on the 5 × 5 × 1 supercell. The dynamic stability was studied by calculating the phonon dispersion using density functional perturbation theory implemented in VASP with a higher electronic convergence criterion of 10^−8^ eV and analyzed by using the PHONOPY code^46^. In addition, dipole correction was applied throughout the calculations^47^. It is noted that based on these settings, the calculated lattice constant (a = 5.18 Å) and band gap (8.6 eV) of *α*-Al_2_O_3_ bulk are in good agreement with previous studies and experimental results^48^,^49^.

## Results and Discussions

### Structural Properties

**Figure 1:**
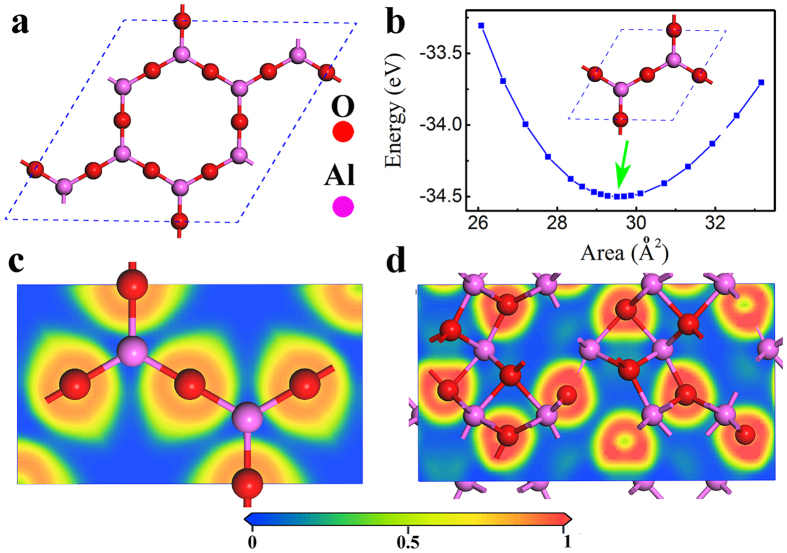
(**a**) Top view of the most stable configuration of Al_2_O_3_ monolayer from the PSO prediction. (**b**) Equation of states of the Al_2_O_3_ monolayer, in which the inset is the top view of a unit cell for the Al_2_O_3_ monolayer. Contour plot of the ELF along Al-O bonding plane for (**c**) Al_2_O_3_ monolayer and (**d**) *α*-Al_2_O_3_ bulk.

The most energetically stable structure of Al_2_O_3_ monolayer predicted by the PSO simulation is shown in Fig. 1(a), from which we can see that the planar 2D structure consists of honeycomb lattices, similar to *h*-BN monolayer and graphene. This honeycomb structure is a global minimum in the space of all possible 2D arrangements of Al_2_O_3_. Then, the predicted Al_2_O_3_ monolayer structure was optimized using first-principle calculations. The equilibrium configuration of Al_2_O_3_ monolayer can be obtained from the energy minimum in the equation of state curve in Fig. 1(b). There are two Al atoms and three O atoms in a unit cell of the Al_2_O_3_ monolayer with the optimized lattice constant of 5.84 Å and the Al-O bond length of 1.69 Å (see the inset in Fig. 1(b)). This bond length is at least 0.18 Å smaller than that of *α*-Al_2_O_3_ bulk. The shorter bond length and variation in bond angle suggest that the in-plane bonding is enhanced in Al_2_O_3_ monolayer in order to suppress surface polarization due to the reduced coordinations, similar to that in Y_2_O_3_ monolayer^25^.

To investigate the bonding character of the Al_2_O_3_ monolayer, we calculated the electron localization function (ELF)^50^, and compared it with that of *α*-Al_2_O_3_ bulk. The contour plot of ELFs for the Al_2_O_3_ monolayer and *α*-Al_2_O_3_ bulk are visualized along the Al-O bond plane, as shown in Fig. 1(c,d), respectively. We observe that the electron density is more localized on the O atoms in Al_2_O_3_ bulk, suggesting dominant ionic bonding character. In contrast, the electron density in the Al_2_O_3_ monolayer is slightly more delocalized. This indicates that the ionic bonding is still dominant in the Al_2_O_3_ monolayer, but it is weaker than that of *α*-Al_2_O_3_ bulk, consistent with the shorter bond length in the Al_2_O_3_ monolayer. The enhanced in-plane covalent bonding character can be seen also from the Bader charge analysis^51^, which suggests an ionic formula of 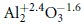 for the Al_2_O_3_ monolayer, less than 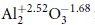 in bulk form.

The stability of a material is indicated by the related cohesive energy, and a structure with negative cohesive energy is stable or metastable. For Al_2_O_3_ monolayer, its cohesive energy *E*_*c*_ can be calculated by the following equation:


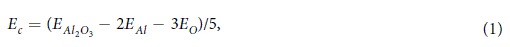


where 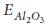 is the total energy of Al_2_O_3_ monolayer, and *E*_*Al*_ and *E*_*O*_ are the energy of isolated single Al and O atom, respectively. The calculated cohesive energy of Al_2_O_3_ monolayer is −5.97 eV/atom, which is slightly higher than that of the Al_2_O_3_ bulk (−6.56 eV/atom), but it is lower than that of recent experimentally confirmed 2D materials such as silicene (−5.16 eV/atom) and germanene (−4.15 eV/atom)^19^,^23^. This indicates that single layer Al_2_O_3_ is promising for an experimental realization.

### Dynamical and Thermal Stability

**Figure 2:**
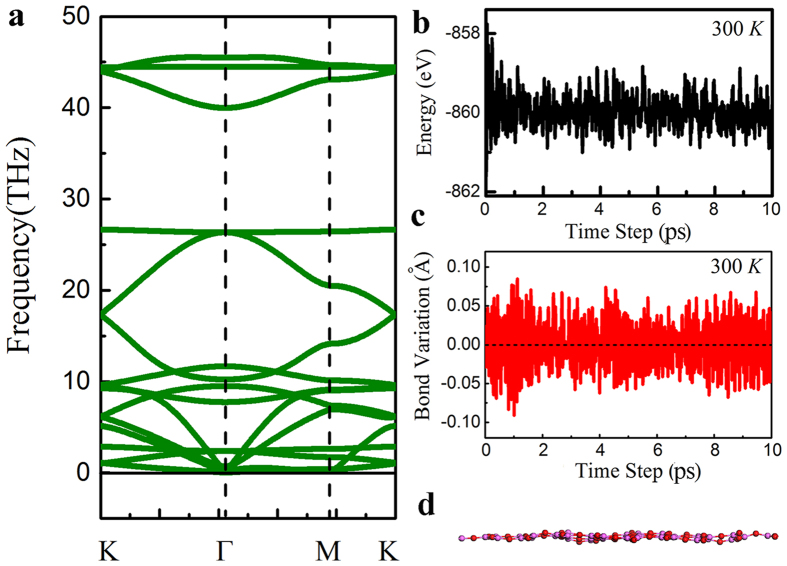
(**a**) Phonon dispersion of the planar Al_2_O_3_ monolayer. (**b**) The energy evolution and (**c**) the evolution of Al-O bond during the 10 ps MD simulation at room temperature, in which the bond variation is defined as the difference between the Al-O bond length in MD simulations and its equilibrium bond length. (**d**) The side view for the Al_2_O_3_ monolayer with the maximum bond displacement during 10 ps the MD simulation.

The structural stability of the Al_2_O_3_ monolayer can also be examined from the calculated phonon spectra. As shown in Fig. 2(a), no negative vibration frequency is found in the phonon dispersion curve in the whole Brillouin zone, indicating that Al_2_O_3_ monolayer is dynamically stable and can exist as a free standing 2D structure.

To further confirm the stability of the Al_2_O_3_ monolayer, *ab initio* molecular dynamics simulation has been conducted at the temperature of 300 K with a time length of 10 ps. A (5 × 5 × 1) supercell of the Al_2_O_3_ monolayer is used to minimize constraints induced by periodic boundary condition. The averaged total energy of the monolayer is nearly constant, and the variation of the Al-O bond length is within 0.1 Å with respect to the average value at 300 K during the simulated time period, as shown in Fig. 2(b,c). Furthermore, even though there is small distortion, the structure remains essentially 2D form after 10 ps (Fig. 2(d)). All these results obtained from the MD simulations suggest that the Al_2_O_3_ monolayer is thermally stable at room temperature. *Ab initio* MD simulations were also carried out at higher temperatures of 800 and 1100 K, respectively, which show that this monolayer is still stable at the temperature of 1100 K (see Fig. S1 in supplementary information).

### Mechanical Properties

Next, we studied mechanical properties such as elastic constant, Young’s modulus, and Poisson’s ratio of the Al_2_O_3_ monolayer. The elastic constant of the Al_2_O_3_ monolayer is calculated by applying the Lagrangian strain (varying the surface area from −3% to 3% deviation in steps of 0.5%). For the deformed state of Al_2_O_3_ monolayer, it is assumed that the contribution of bending to the strain energy density is insignificant, thus the vertical weight of elastic constants is zero. For all the deformed configurations, the positions of all atoms were optimized and the related energies were calculated. In this way, for each type of distortion, we could obtain the strain dependent energy. Then, the in-plane Young’s modulus *Y*_*s*_ and Poison’s ratio *v* were calculated from the following relationship: 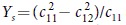 and 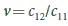, respectively. For a comparison, the mechanical properties of graphene, *h-*BN monolayer and ZnO monolayer were calculated and summarized in Table 1. The mechanical parameters of graphene, BN and ZnO monolayer obtained in the present study are in good agreement with results of previous studies. The Young’s modulus of the Al_2_O_3_ monolayer is 47.6 N/m, very close to that of ZnO monolayer (~47 N/m), but much smaller than that of graphene (~342 N/m), BN monolayer (~274 N/m), and Al_2_O_3_ bulk (~230 GPa). This clearly shows the relative softness of the Al_2_O_3_ monolayer. The softness of the Al_2_O_3_ monolayer is also reflected by the flat slope of the acoustic branch at the Γ point in the phonon dispersion^52^, as shown in Fig. 2(a). These indicate that Al_2_O_3_ monolayer is susceptible to external influence, with greater tendency to form ripples or out-of-plane deformations, as supported by the above MD simulation results (see Fig. 2(d)).

### Electronic Properties

**Figure 3:**
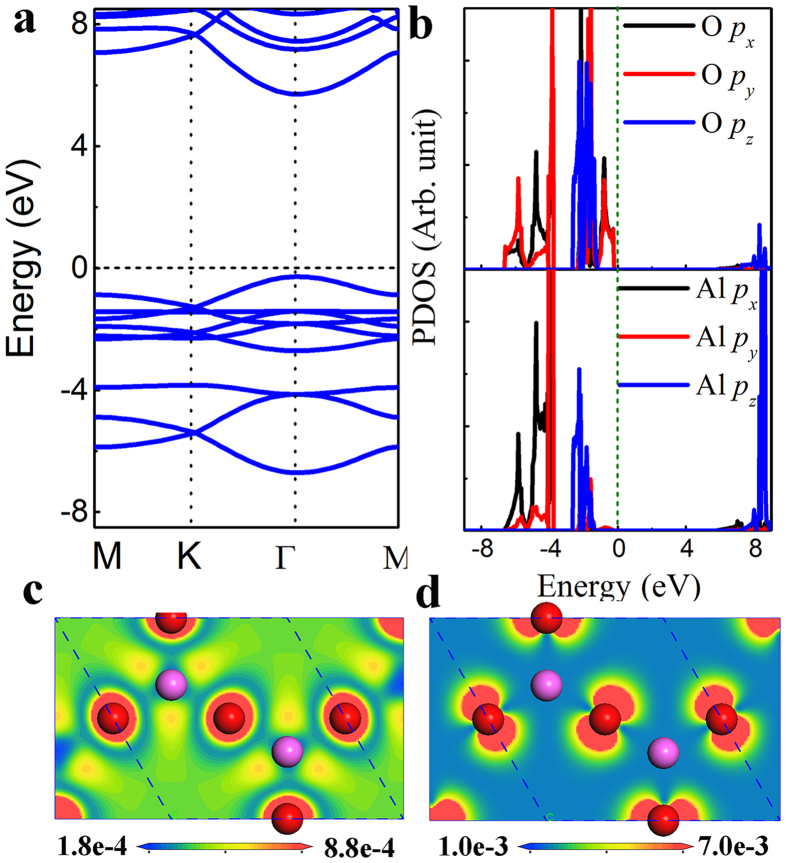
(**a**) The HSE06 hybrid functionals calculated band structure and (**b**) the projected density of states of the Al_2_O_3_ (111) monolayer. The Fermi level is shifted to 0 eV. The contour plot of partial charge density (**c**) near the conduction band minimum and (**d**) near the valence band maximum for the Al_2_O_3_ (111) monolayer.

Al_2_O_3_ bulk is an excellent dielectric material, which has been widely applied in current electronic devices. One natural question is whether it is a promising dielectric if its thickness is reduced to its physical limit, one monolayer. One of the most important criteria for a dielectric material is the size of the band gap. A dielectric material with large band gap is desired as it can reduce the probability of thermal carrier tunneling. The HSE06 hybrid functionals calculated band structure and projected density of states of the Al_2_O_3_ monolayer are shown in Fig. 3(a,b), which suggest a direct band gap of 5.99 eV at the Γ point. For comparison, the band structure of *α*-Al_2_O_3_ was calculated also and presented in Fig. S2 of the supplementary information, in which a direct band gap of 8.6 eV is found. Due to the change of bonding character from the bulk to monolayer and also quantum confinement effect, the band gap of the Al_2_O_3_ monolayer is much reduced, but comparable to that of HfO_2_ (~5.7 eV), one of the most promising high-*κ* dielectrics. Thus, we believe that the band gap of the Al_2_O_3_ monolayer is large enough to be used as a dielectric material. From the PDOS and partial charge density (see Fig. 3(c,d)), we find that the main contributions to the valence band maximum come from the *p*_*x*_ and *p*_*y*_ orbitals of the O atoms, and the conduction band minimum is derived from the hybridization between O and Al 2 *p* orbitals. This further confirms dominant ionic bonding character in Al_2_O_3_ monolayer, in line with the above ionic formula obtained by the Bader charge analysis. At the energy range from −3 eV to −1 eV, the formation of *π* bonds is found as the *p*_*z*_ orbitals of O atoms and Al atoms hybridize with each other, as well as *σ* bonds (the hybridization of *p*_*x*_ or *p*_*y*_ orbitals between O and Al atoms). The formation of *π* bonds is believed favorable to stabilize the 2D planar structure, as shown in other 2D structures^53^.

### Dielectric Properties

**Figure 4:**
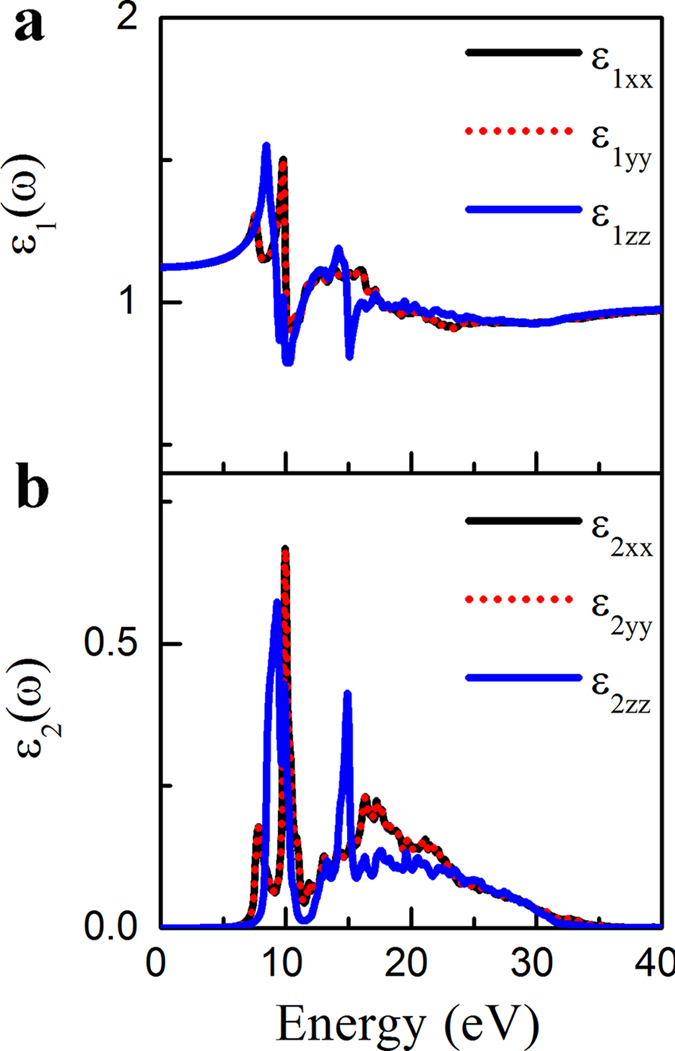
The HSE06 hybrid functionals calculated dielectric function of the Al_2_O_3_ monolayer: (**a**) real part and (**b**) imaginary part.

A dielectric material with high dielectric constant is preferred in order to achieve larger capacitance density. The dielectric constant decreases with the decrease of thickness, and thus it is important to study dielectric properties for a dielectric material with one atomic thickness. The calculated dielectric function of the Al_2_O_3_ monolayer is shown in Fig. 4(a,b). Due to hexagonal symmetry of the structure, the imaginary and real parts of the dielectric function are degenerate along the x and y axes. Thus, for these two independent sets of dielectric tensors, *ε*_*xx*_ and *ε*_*zz*_, the total dielectric constant *ε* can be determined by 
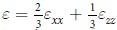
.

The electronic contribution of the static dielectric constant for the Al_2_O_3_ monolayer is determined by the zero energy limit from the real dielectric function, which is about 1.27. With the inclusion of the ionic vibration contribution (~1.24), the overall dielectric constant is about 2.51. This value is much smaller than that of *α*-Al_2_O_3_ bulk (~10.5) due to the reduced thickness, changed bonding character, and also decreased band gap, but comparable to that of SiO_2_ bulk (~3.9). It is noted that the calculated dielectric constant of *α*-Al_2_O_3_ bulk is consistent with the experiment value (~9.0). From the imaginary part the dielectric function (Fig. 4(b)), we can see that the main adsorption peak locates at 10 eV, which is mainly contributed by the inter-band transition from O *p*_*z*_ orbitals to Al *p*_*z*_ orbitals. Moreover, the adsorption edge is at the energy of around 6 eV, in good agreement with the calculated band gap.

### The Interface between Graphene and Al_2_O_3_ Monolayer

Experimentally, various substrates have been proposed for growing oxide monolayer such as SiO_2_ and Y_2_O_3_ monolayer, in which substrates with inert surface are desired^23^,^34^. It is noted that Y_2_O_3_ monolayer has been deposited on graphene substrate^34^, which motivated us to study the feasibility of using graphene as a substrate to grow Al_2_O_3_ monolayer. Therefore, the interfacial properties of graphene and Al_2_O_3_ monolayer are studied also. We find that (4 × 4) graphene supercell matches 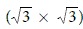 Al_2_O_3_ monolayer well. Since the Al_2_O_3_ monolayer is relatively soft, it was compressed by about 2.88% and placed on graphene supercell. This small strain on the Al_2_O_3_ monolayer yields a 0.18 eV increase in the band gap, but does not alter the electronic properties significantly, as shown in the Fig. S3 in the supplementary information. It is also noted that with the presence of graphene substrate, the Al_2_O_3_ monolayer still favors planar structure, and becomes more stable as its energy is lowered by 0.19 eV per unit cell, compared with free standing Al_2_O_3_ monolayer.

**Figure 5:**
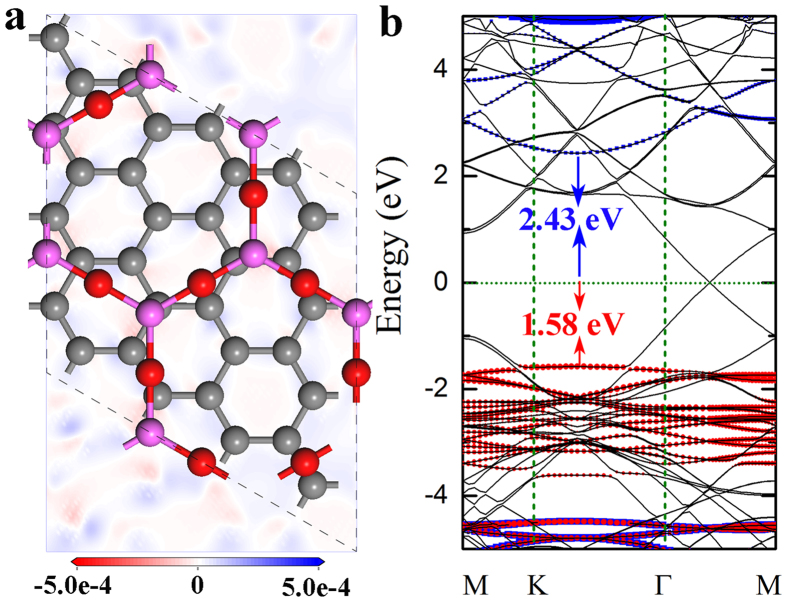
(**a**) Top view of the most stable interface structure of the Al_2_O_3_ monolayer on graphene imposed with contour plot charge density difference projected on the graphene plane. The blue color denotes the excess charge, and the red color denotes the depleted charge. (**b**) The band structure of the Al_2_O_3_ monolayer on graphene, where the black lines denote the contribution from graphene, the red solid dots denote the contribution from O atoms, and the blue square lattices denote the contribution from Al atom. The Fermi energy is shifted to 0 eV.

The most stable configuration of the Al_2_O_3_ monolayer on graphene is shown in Fig. 5(a), where the Al atoms are located near the top sites of C atoms in graphene in order to form maximized potential local bonding. This is similar to that of Y_2_O_3_ monolayer, *β*-Si_3_N_4_, and g-C_3_N_3_ on graphene, and HfO_2_ on MoS_2_ monolayer^25^,^54^,^55^,^56^,^57^. After relaxation, both graphene and the Al_2_O_3_ monolayer planes remain planar. The shortest distance between Al_2_O_3_ monolayer and graphene is 3.26 Å, indicating weak interaction between them. The calculated adsorption energy is 13.62 meV/Å^2^, smaller than that of graphene on Al_2_O_3_ (0001) surface^58^, which confirms the weak interfacial interaction. Bader charge analysis suggests that there is no interfacial charge transfer between the Al_2_O_3_ monolayer and graphene.

Due to the weak interaction, the charge inhomogeneity in graphene is insignificant with the presence of the Al_2_O_3_ monolayer. From the charge density difference projected on graphene plane (Fig. 5(a)), we can see that the charge inhomogeneity is below 5 × 10^−4^ e/Å^2^. This indicates that the electron-hole puddles are negligible in graphene. The electron-hole puddles on channel materials are undesired because they can reduce the carrier mobility^59^. The band structure of the Al_2_O_3_ monolayer/graphene interface is shown in Fig. 5(b). It is observed that the linear electronic dispersion in graphene is nearly intact due to the weak interfacial interaction. The valence band edge of the Al_2_O_3_ monolayer locates at the energy of −1.58 eV, which is mainly from the contribution of O 2*p* orbitals. The conduction band edge of the Al_2_O_3_ monolayer is at the energy of 2.43 eV, and the dominant contribution is from the hybridization of O *p* and Al *p* orbitals. If we treat graphene as a semiconductor with 0 eV band gap, the band alignment between graphene and the Al_2_O_3_ monolayer can be estimated from their relative band edge shift, which is 1.58 and 2.43 eV for valence (VBO) and conduction band offset (CBO), respectively, as shown in Fig. 5(b). Both the VBO and CBO are larger than 1 eV, large enough for potential electronic applications. We wish to point out that PBE functionals were used in this calculation, which leads to an underestimated band gap of the Al_2_O_3_ monolayer. Thus, the real VBO and CBO should be larger than the values in current studies.

From the above discussions, we can see that graphene has same surface symmetry, small lattice mismatch, and weak interaction with Al_2_O_3_ monolayer, thus it is a potential substrate to grow Al_2_O_3_ monolayer, similar to the growth of Y_2_O_3_ monolayer on graphene^34^. One common way for the growth of metal oxide thin films is to thermally oxidize the metal surface directly. In order to explore the feasibility of this growth strategy on the Al_2_O_3_ monolayer, the interface between the Al_2_O_3_ monolayer and Al (111) surface has been studied also (see the details in the supplementary information). It turns out that the interaction between Al (111) and the Al_2_O_3_ monolayer is strong. After relaxation, the most energetically favorable configuration is that the O atoms in the Al_2_O_3_ monolayer move toward the Al (111), forming interfacial Al-O bonds with bond length of 1.9 Å. The strong interaction is also confirmed by the large adsorption energy (2.2 eV per Al_2_O_3_ unit) and significant charge transfer (see Fig. S4 in the supplementary information). This strong interaction leads to a buckled structure of Al_2_O_3_ monolayer on Al (111), and it can be expected that the 2D growth of Al_2_O_3_ monolayer is difficult on Al (111) substrate by a direct thermal oxidation process. Therefore, inert substrates with weak interaction, same surface symmetry, and small lattice mismatch such as graphene are favored to realize the 2D growth of Al_2_O_3_ monolayer *via* sputtering or other advanced deposition techniques.

## Conclusions

In conclusion, based on PSO method and first-principles calculations, we find a stable Al_2_O_3_ monolayer with a direct band gap of 5.99 eV and dielectric constant of 2.51. This Al_2_O_3_ monolayer can be further stabilized by the support of graphene. For the interface between Al_2_O_3_ monolayer and graphene, we find that the interaction between them is weak and band alignments are much larger than 1 eV. Moreover, the presence of the Al_2_O_3_ monolayer does not introduce interface states and electron-hole puddles in graphene. Together with high thermal stability, the Al_2_O_3_ monolayer might be a potential dielectric for future electronic applications. Due to similar surface chemistry between graphene and other 2D channel materials such as transposition metal dichalcogenide or phosphene, these results might be applicable to the interface between the Al_2_O_3_ monolayer and other 2D materials.

## Additional Information

**How to cite this article**: Song, T. T. *et al*. The stability of aluminium oxide monolayer and its interface with two-dimensional materials. *Sci. Rep.* **6**, 29221; doi: 10.1038/srep29221 (2016).

## Supplementary Material

Supplementary Information

## Figures and Tables

**Table 1:** The elastic constant, Young’s modulus, and Poisson’s ratio of Al_2_O_3_ monolayer, graphene, BN monolayer, and ZnO monolayer.

	*c*_11_ (N/m)	*c*_12_ (N/m)	*Y*_*s*_ (N/m)	*v*	
Al_2_O_3_ monolayer	87.75	59.35	47.6	0.676	this work
Graphene	353.675	62.195	342.7	0.176	this work
	352	62.6	340.8	0.178	ref. 60
	353	61	342	0.17	ref. 61
	358.1	60.4	348	0.17	ref. 62
BN-monolayer	288.26	63.54	274.3	0.22	this work
	293.2	66.1	278.3	0.225	ref. 60
	290	64	276	0.22	ref. 61
ZnO monolayer	85.34	56.54	47.88	0.663	this work
	86	57.3	47.8	0.667	ref. 61
