# Supplementary material for: The stability of aluminium oxide monolayer and its interface with two-dimensional materials
Source: Sci Rep. 2016 Jul 6;6:29221. doi: 10.1038/srep29221 (PMC4933898; doi:10.1038/srep29221)
Supplement: Supplementary Information [file srep29221-s1.pdf]

Supplemental Information for

The stability of aluminium oxide monolayer and its interface with two-dimensional materials

Ting Ting Song[1], Ming Yang[2,4†,] Jian Wei Chai[2], Martin Callsen[3], Jun Zhou[3], Tong Yang[2,4], Zheng Zhang[2], Ji Sheng Pan[2], Dong Zhi Chi[2], Yuan Ping Feng[3], and Shi Jie Wang[2,‡]

[1] Institute for Structure and Function and Department of Physics, Chongqing University, Chongqing, People's Republic of China 400044.

[2] Institute of Materials Research and Engineering, 2 Fusionopolis Way, Singapore 138634.

[3] Department of Physics, National University of Singapore, 2 Science Drive 3, Singapore 117542.

[4] Centre for Advanced 2D Materials and Graphene Research, National University of Singapore, 6 Science Drive 2, Singapore 117546.

*Correspondence and requests for materials should be addressed to M. Y[†] (e-mail: yangm@imre.a-star.edu.sg) or S. J. W[‡] (e-mail: sj-wang@imre.a-star.edu.sg)

1. MD simulation results at the temperature of 1100 *K*

The thermal stability of the Al_2O_3 monolayer is also examined at a high temperature of 1100 *K* using *ab initio* quantum molecular dynamics simulation, as shown in Fig. S1. It is noted that the averaged variation of total energy of the Al_2O_3 monolayer is nearly constant for the simulated 10 *ps* time step. The variation of the bond length is within 0.2 Å, slight larger than that in 300 *K*. The top and side view of the Al_2O_3 monolayer after 10 *ps* time step is shown in Fig. S1(c) and (d), respectively, which clearly show that the Al_2O_3 monolayer remains stable at the temperature of 1100 K. The distortion of in-plane lattice is insignificant, but the

distortion of out-of-plane is much more profound, compared with that at simulated temperature of 300 *K*.

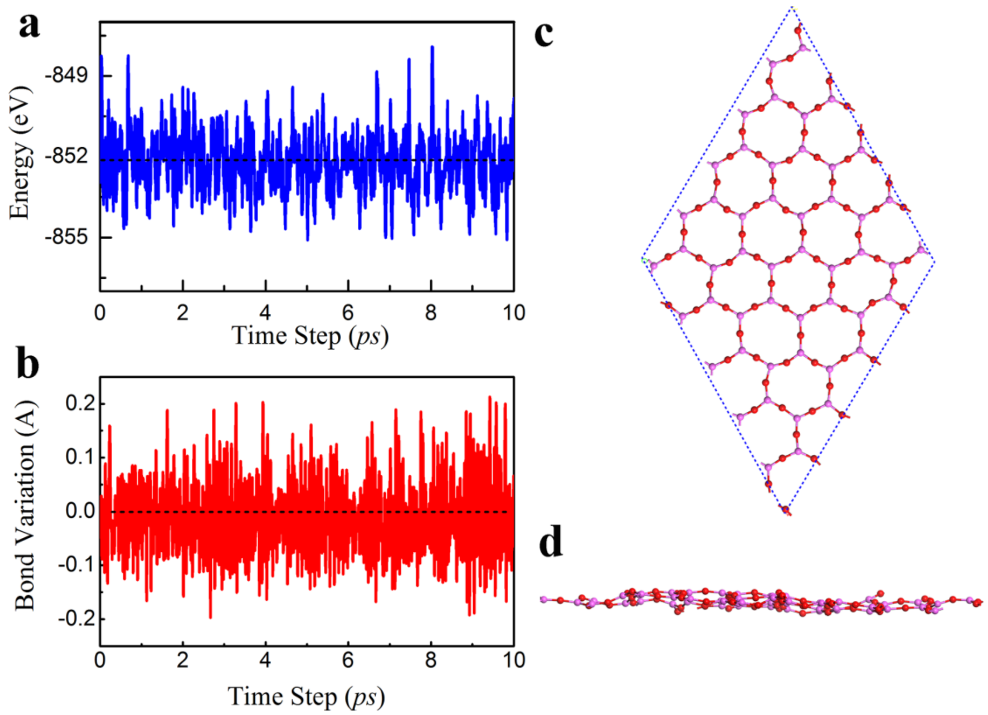

Figure S1: MD simulation of the Al_2O_3 monolayer at the temperature of 1100 *K*. (a) The energy evolution and (b) the variation of bond length of the Al_2O_3 monolayer at the 10 *ps* time steps. (c) The top view and (d) side view of the Al_2O_3 monolayer after 10 *ps* time steps.

2. Band structure of α-Al_2O_3 bulk

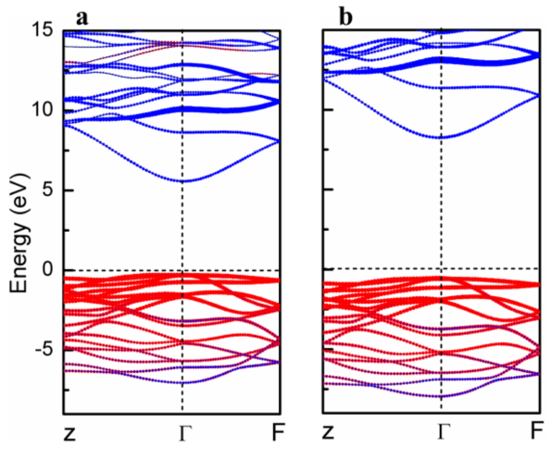

Figure S2: PBE (a) and HSE06 (b) hybrid functionals calculation band structure of α-Al_2O_3, where the blue and red dots show the contribution from the p orbitals of Al and O atoms, respectively. The Fermi level is shifted to 0 eV.

The PBE and HSE06 hybrid functionals calculated band structure of α-Al_2O_3 bulk is shown in Fig. S2(a) and (b), respectively. The α-Al_2O_3 bulk is a direct band gap insulator with a PBE band gap of 5.81, in good agreement with previous study.[49] The HSE06 hybrid functionals calculations give a much improved band gap of 8.6 eV, which is close to experimental value.

3. Electronic properties of the Al_2O_3 monolayer under the compressive strain

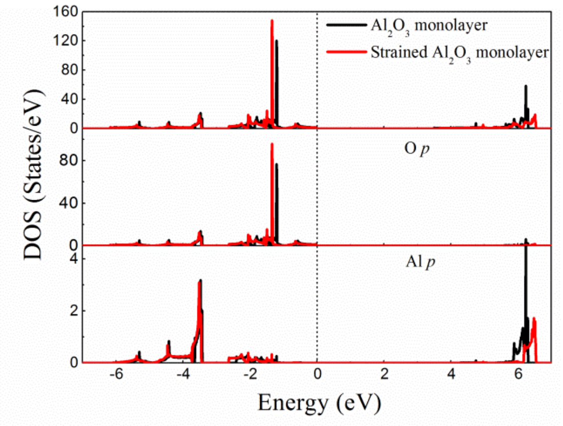

Figure S3: Density of states and partial density of states of Al_2O_3 monolayer with and without 2.88% compressive strain. The Fermi level is shifted to 0 eV.

The PBE functional calculated density of states of the Al_2O_3 monolayer with and without the 2.88% biaxial compressive strain is shown in Fig. S3. It is noted that the compressive strain leads to a band gap increase of 0.18 eV, but the strain does not alter the electronic properties significantly. The conduction band maximum of the strained Al_2O_3 monolayer is mainly contributed by the O *p* orbital, and the conduction band minimum is derived from the hybridization between O *p* and Al *p* orbital, which is the same as those in the Al_2O_3 monolayer without strain.

4. The interface between Al_2O_3 monolayer and Al (111) substrate

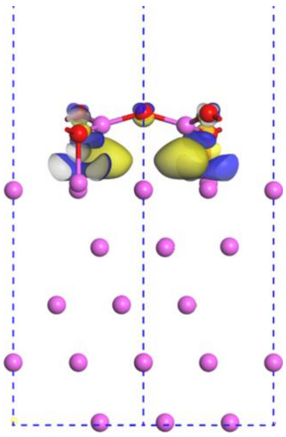

Figure S4: The most stable structure of the Al_2O_3 monolayer on the Al (111) substrate with the imposed charge density difference. The blue color denotes charge accumulation, and the yellow color denotes charge depletion. The charge density difference is visualized with an isosurface value of 1.0×10^{-3} e/A^3 (side view).

For the interface between the Al_2O_3 monolayer and Al (111) substrate, it is noted the (1×1) Al_2O_3 monolayer matches well with (2×2) Al (111) surface, in which the Al_2O_3 monolayer was compressed about 2.2% and placed on five layers of Al (111) substrate. The most stable structure is that O atoms of the Al_2O_3 monolayer on the top of Al atoms in Al (111) substrate. After relaxation, the O atoms move toward the Al substrate, and form interfacial Al-O bonds with a bond length of 1.91 Å, close to that in Al_2O_3 bulk. This leads to a buckled Al_2O_3 monolayer, as shown in Fig. S4.

The interaction between the Al_2O_3 monolayer and Al (111) is found strong. The calculated adsorption energy is about 2.2 eV per Al_2O_3 unit, indicating that the interaction is chemisorption between the Al_2O_3 monolayer and Al (111). The strong interaction also results in significant interfacial charge transfer. Bader charge analysis shows that there is about 0.4 electrons transferred from the Al substrate into the Al_2O_3 monolayer. This suggests a more polarized Al_2O_3 monolayer. The strong interaction, buckled structure and polarized Al_2O_3 monolayer on the Al (111) substrate infer that it is difficult to realize 2D growth of the Al_2O_3 thin film.
